# The structural stability, electronic properties regulation and feasibility of controllable preparation of a C_0.5_/(BN)_0.5_ heterojunction single-walled nanotube

**DOI:** 10.1016/j.heliyon.2023.e19382

**Published:** 2023-08-25

**Authors:** Feiyu Zhu, Yanbo Zou, Junzhe Lu, Jie Wei, Hengjiang Zhu

**Affiliations:** a Xinjiang Key Laboratory for Luminescence Minerals and Optical Functional Materials, School of Physics and Electronic Engineering, Xinjiang Normal University, Urumqi, Xinjiang 830054, China; b Laboratory and Equipment Management Division, Xinjiang Normal University, Urumqi, Xinjiang 830054, China

**Keywords:** Heterojunction single-walled nanotubes, Structural stability, Electronic property regulation, Controllable preparation

## Abstract

Our work investigates the structural stability of a $C_{0.5}/{\left(\text{BN}\right)}_{0.5}$ heterojunction single-walled nanotube by comparing the binding energy. The energy band structure, electronic density of states and regulation relation between band gap and indirect-direct properties and tube diameter and type are systematically studied. Based on existing experimental and theoretical results, dynamic simulated calculating of the stitching process is carried out to explore the feasibility of controllable preparation.

## 1 Introduction

Carbon nanotubes (CNTs) have a wide application range due to their excellent properties, such as high carrier mobility, high light transmission, high tensile strength and low electrical resistance [[1], [2], [3], [4], [5], [6], [7], [8]]. However, depending on their type (chiral angle), tube diameter and number of walls, CNTs' electronic properties can be metallic conductors or semiconductors with varying band gaps. This is supposed to be an advantage in the electronic properties of carbon nanotubes. However, since CNTs were observed under an electron microscope by Japanese scientist Sumio Ijima in 1991, CNTs prepared by various methods are often a mixture of single- and multi-walled. In which case, the single-walled CNTs (SWCNTs) are usually also a mixture of different types and tube diameters. Results show that the band gap of semiconducting SWCNTs decreases with tube diameter increasing and the band gap is adjustable between 0.5 and 1.2eV in the 0.7–2.0 nm limit of tube diameter [9]. For more than 30 years, scientists from different countries have been searching for effective ways to prepare CNTs of controlled type and tube diameter (selective or customized), with the hope of obtaining CNTs of a single electronic character for their wide ranging and effective applications. Professor Li's group [10,11] proposed a growth scheme to control the type and tube diameter of SWCNTs, and higher purity single-electron SWCNTs such as 92% (12,6) and 79.2% (16,0) were successfully prepared, which strongly enhanced the theoretical and experimental research on the controllable preparation of single-electron SWCNTs. However, the problem of controllable preparation of CNTs with a single electronic character has not been completely solved, which greatly restricts the application of CNTs in modern optoelectronic and electronic technology. For example, M. M. Schulak and others successfully fabricated no silicon-based chips from semiconducting CNTs with a purity of 99.99% after secondary purification, indicating that chip manufacturing is likely to have a new milestone development. However, unfortunately due to the non-100% purity of semiconducting CNTs, only a single transistor can be prepared using multiple CNTs as the channel material, which leads to an overly wide transistor gate, increasing the geometric scale of the transistor. In addition, the band gap of semiconductor CNTs with large diameter macro prepared is generally small at present, which limits the operating voltage, resulting in low transistor power and reduced speed. Ultimately, the size and speed of CNTs are not better than those of commercial silicon-based chips [12].

Boron nitride nanotubes (BNNTs) are one of the nanotubes that have been intensively studied and which achieved good results after CNTs [[13], [14], [15], [16], [17], [18], [19]]. As with CNTs, BNNTs have many excellent properties, such as high hardness, and high thermal and chemical stability, making them excellent insulating and thermal insulating materials for a wide range of applications, such as in composite materials, hydrogen storage and force sensors [[20], [21], [22], [23]]. In addition, BNNTs’ best characteristic in terms of electronic properties is an ultra-wide band gap of about 5.9eV [24,25], close to that of an insulator [26], and largely independent of type, tube diameter and wall number [27]. However, the ultra-wide band gap also limits the direct application of BNNTs' excellent properties in areas such as nanoscale semiconductor optical and electrical components. Therefore, reducing the band gap width, while retaining its excellent performance, has become a real need in order to expand the application of BNNTs in nanoscale semiconductor optical and electrical devices. Currently, the main methods of reducing the band gap width of BNNTs are as follows: strain stress (0.25–3.60eV), doping [28] (3.98–4.65eV), nitrobenzene derivatives instead of adsorption [29] (2.69–3.45eV) and structural reconstruction (2.530–4.001eV) [30]. Among them, the adsorption and structural reconstruction methods are best for band gap reduction, as they meet the band gap requirements of third generation semiconductors (wide band gap semiconductors (﹥2.0eV)) [31].

Heterojunctions are regions formed by the contact of two different materials and research into them started with semiconductor heterojunctions. Over the years, extensive and significant research results have been achieved in semiconductor heterojunctions, both theoretically, experimentally and in specific applications. In particular, the invention and subsequent development of the transistor laid the foundations for modern electronics and the information revolution. Heterojunctions are mainly obtained by doping of homogeneous materials, such as PN junctions in semiconductor silicon, or by epitaxial growth on lattice-matched substrate materials.

**Fig. 1:**
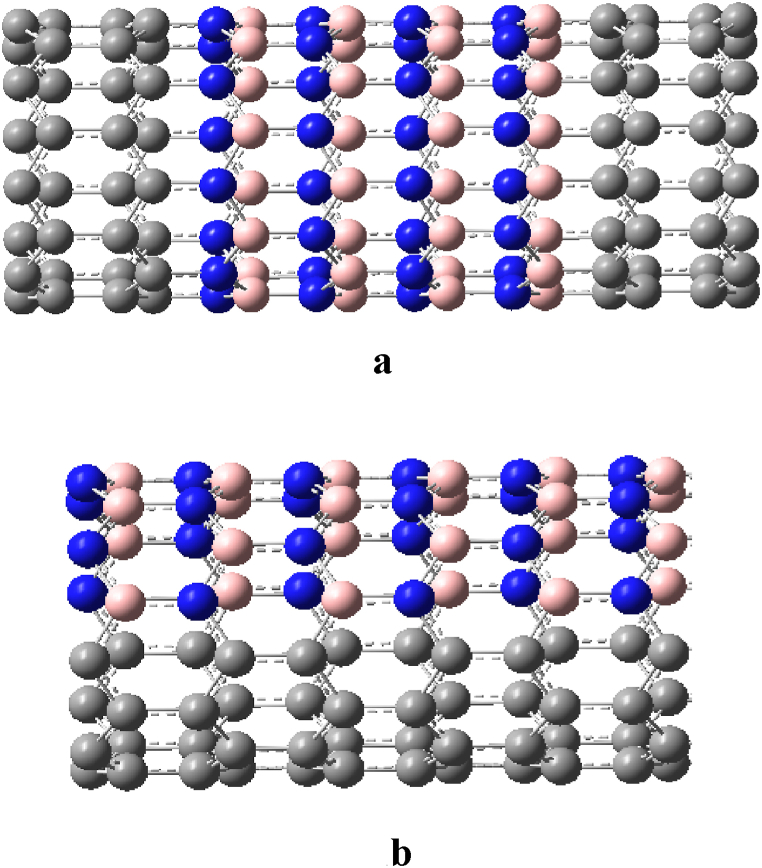
(a) The same type, similar pipe diameters, SWCNTs and SWBNNTs are docked along the cross-section, forming C_4_/(BN)_4_ heterojunction sawtooth SWNTs [36] (b) Part of SWCNTs of the same type and similar tube diameters are buckled with part of SWBNNTs, forming C_0·5_/(BN)_0.5_ heterojunction sawtooth SWNTs [39].

The SWCNTs and single-walled BNNTs (SWBNNTs) are isomorphic structure. They have similar repeating unit length (1.94–3.28%, see Table 1) into they have a good lattice match, they can constitute heterojunction nanotubes. In the literature [[32], [33], [34], [35], [36], [37], [38]], $C_{k}/{\left(\text{BN}\right)}_{q}\left(k,q=1,2,3...\right)$ heterojunction single-walled nanotubes (SWNTs) were constructed by segmentally docking SWCNTs of the same type and similar tube diameter with SWBNNTs (see Fig. 1a). In the literature [[37], [38], [39]], $C_{x}{\left(\text{BN}\right)}_{1-x}\left(0＜x＜1\right)$ heterojunction SWNTs were constructed by snapping together some SWCNTs and some SWBNNTs of the same type and similar tube diameter, and separating C and BN in different proportions along the busbar (see Fig. 1b). In January 2020, the successful preparation of a SWCNT wrapped with a SWBNNT or two layers of BNNTs, a multi-tube nested one-dimensional van der Waals heterogeneous nanotube [40], renewed interest in heterojunction nanotubes formed from C and BN. The aim of designing, researching and preparing heterojunction nanotubes formed by contacting C and BN is to combine the advantages of CNTs and BNNTs, and at the same time to open the band gap of metallic CNTs or increase the band gap of semiconducting CNTs by taking advantage of the ultra-wide band gap of BNNTs, so as obtain nanotubes with a band gap between CNTs and BNNTs and a perfect heterojunction, achieving the effect of “1 + 1 > 2". Although the research on $C_{k}/{\left(\text{BN}\right)}_{q}$ heterojunction SWNTs started early and a series of results have been achieved in both experimental and theoretical studies, such as the successful preparation of various types of $C_{k}/{\left(\text{BN}\right)}_{q}$ heterojunction SWNTs with good modulation of the band gap [[32], [33], [34], [35], [36], [37], [38]] the heterojunction defects are many due to the constraints of the preparation method and other factors, and controlled preparation has not been realized until today. In contrast, $C_{x}{\left(\text{BN}\right)}_{1-x}$ heterojunction SWNTs, due to their structural peculiarities, have not been prepared experimentally and related studies are limited to theoretical studies of both armchair and sawtooth types [[37], [38], [39]]. Therefore, the reasons for the unsuccessful preparation of $C_{x}{\left(\text{BN}\right)}_{1-x}$ heterojunction SWNTs, whether it is the structural instability or the preparation method, as well as what the advantages of the electronic properties of $C_{x}{\left(\text{BN}\right)}_{1-x}$ heterojunction SWNTs are compared with SWCTs and SWBNTs, especially whether their controllable preparation can be achieved, need further theoretical and experimental studies.

**Table 1:** Relative differences in lattice constants (minimum repeat cell length) for SWCNTs and SWBNNTs.

(*n*, *m*)	SWCNTs	SWBNNTs	SWCNTs relative difference	SWBNNTs relative difference
(3, 3)	2.465050	2.546073	0.032868	0.031822
(4, 4)	2.464825	2.538945	0.030071	0.029193
(5, 5)	2.465225	2.535933	0.028682	0.027882
(6, 6)	2.465823	2.534823	0.027982	0.027219
(7, 7)	2.465856	2.533891	0.027590	0.026849
(8, 8)	2.466546	2.533334	0.027076	0.026361
(9, 9)	2.466698	2.532966	0.026862	0.026159
(10, 10)	2.466634	2.532724	0.026792	0.026093
(4, 0)	2.830425	2.900004	0.024581	0.023991
(5, 0)	2.845558	2.901843	0.019779	0.019395
(6, 0)	2.848660	2.911861	0.022186	0.021704
(7, 0)	2.848283	2.916764	0.024041	0.023477
(8, 0)	2.848765	2.919224	0.024733	0.024136
(9, 0)	2.848766	2.921010	0.025359	0.024732
(10, 0)	2.848640	2.921367	0.025530	0.024894
(11, 0)	2.848651	2.921877	0.025705	0.025061
(4, 2)	11.27800	11.62189	0.030492	0.029589
(6, 3)	11.29069	11.61135	0.028401	0.027616
(8 4)	11.30354	11.60728	0.026871	0.026181
(10, 5)	11.30015	11.60506	0.026983	0.026274
(12, 6)	11.30210	11.60387	0.026700	0.026005
(14, 7)	11.30480	11.60307	0.026384	0.025705
(16, 8)	11.30431	11.60181	0.026318	0.025643
(18, 9)	11.30387	11.60141	0.026323	0.025647
(20, 10)	11.30566	11.60133	0.026155	0.025488

## 2 Research content, method and objectives of the study

In this paper, we investigate the structural stability, electronic properties modulation and feasibility of controllable preparation of $C_{0.5}/{\left(\text{BN}\right)}_{0.5}$ heterojunction single-walled nanotubes with different tube diameters in equal proportions using density flooding theory (DFT) based on the first-nature principle. Our group successively investigated the electronic properties and structural derivation of CNTs and BNNTs using the G03 commercial software package and selected the B3LYP/3-21 g generalized function/base set [[41], [42], [43], [44], [45]], respectively, and obtained results consistent with the relevant experiments. The B3LYP generalized function is a widely used hybridization density function which employs three different functions, namely Becke's three-parameter exchange-correlation function, Lee, Yang and Parr's local density approximation function, and Vosko, Wilk and Nusair's nonlocal exchange-correlation function. The 3–21 g basis group uses a non-local treatment for the extra-nuclear electrons, i.e., global considerations. As a result, the results of their simulations tend to be highly reliable. In particular, the inner electrons are represented by STO-3G, and the outer first and second valence layers are represented by STO-2G and STO-1G, respectively, for the split valence layer basis set. Such a split valence layer increases the flexibility for the radial description of the wavefunction and allows a more accurate calculation of the global charge. Therefore, this paper continues to use the G03 commercial software package, select the B3LYP/3-21 g generalized function/base group, adopt periodic boundary conditions (PBC) (without changing the polarity of the nanotubes), the electronic energy state is a single heavy state and the energy harvesting criterion is 10^−6^a.u. In this work, we carry out structural optimization calculations for three types of $C_{0.5}/{\left(\text{BN}\right)}_{0.5}$ heterojunction SWNTs, and study their structural stability by combining energy comparisons. Then we calculate the energy band structure and electron density of states, and to investigate more comprehensively the regulatory relationships between band gap and interstitial, direct properties and type and tube diameter. Finally, based on the existing experimental and theoretical research results, the feasibility of their controllable preparation is explored through dynamic mapping calculations. The aim of the research is to develop a nanoscale material that combines the advantages of SWCNTs and SWBNNTs, with a band gap between SWCNTs and SWBNNTs, a perfect heterojunction, and well-tuned electronic properties, in order to provide a high-quality material for the fabrication of nanoscale optical and electrical components and chips. Secondly, we investigate theoretically whether it can be prepared in a controlled manner with the electronic properties required.

As with SWCNTs and SWBNNTs, $C_{0.5}/{\left(\text{BN}\right)}_{0.5}$ heterojunction SWNTs can still be represented by (n,m) (n,m=0, 1, 2 …). When n=m≠0, (n,n) has no chirality it is said to be armchair; when n=0≠m or m=0≠n, (0,m) or (n,0) have no chirality and both are called zigzag; when n≠m and n≠0≠m, (n,m) has chirality and is said to be chiral. The chiral vector of (n,m) is defined as:

(1) $${\vec{C}}_{nm}=n{\vec{a}}_{1}+m{\vec{a}}_{2}$$

In equation (1), the ${\vec{a}}_{1}$ and ${\vec{a}}_{2}$ are the basis vectors for the angular 60° oblique coordinate system.

## 3 Results and analysis

### 3.1 Structure and structural stability

#### 3.1.1 Structure

Table 2 shows the average bond lengths of SWCNTs, SWBNNTs and $C_{0.5}/{\left(\text{BN}\right)}_{0.5}$ heterojunction SWNTs. The results show that for the same *n* and *m*, the average *C*–C bond length in $C_{0.5}/{\left(\text{BN}\right)}_{0.5}$ heterojunction SWNTs is slightly larger than that of *C*–C in SWCNTs, whereas the average B–N bond length is slightly smaller than the average B–N bond length in SWBNNTs (in agreement with the conclusion of Ref. 38), especially the *C*–N bond at the heterojunction is slightly smaller than its own *C*–C bond, while the *C*–B bond is larger than its own *C*–C bond, which ensures the lattice matching requirement for the coexistence of C and BN in the same tube. On the other hand, Table 3a, Table 3ba, 3b shows that for the same *n* and *m*, the average bond angles of all three nanotubes are less than 120° due to tube surface curvature effects, and the average bond angles converge to 120° with increasing tube diameter, i.e., *n* or *m*, indicating that, like the SWCNTs and SWBNNTs, the $C_{0.5}/{\left(\text{BN}\right)}_{0.5}$ heterojunction SWNTs are still *sp*^2^ orbital hybridization.

**Table 2:** Average bond length.

(*n*, *m*)	SWCNTs	SWBNNTs	C_0·5_/(BN)_0.5_ heterojunction SWNTs				
	C–C	B–N	C–C	B–N	C–N	C–B	Average bond length^╂^
(3, 3)	1.43858	1.47534	1.44708	1.46377	1.42348	1.54615	1.45934
(4, 4)	1.43245	1.47023	1.44098	1.45809	1.41491	1.54028	1.45328
(5, 5)	1.42967	1.46750	1.43585	1.45507	1.41142	1.53971	1.44947
(6, 6)	1.42826	1.46623	1.42855	1.46202	1.42311	1.54331	1.45034
(7, 7)	1.42696	1.46515	1.42895	1.46012	1.42122	1.54431	1.44963
(8, 8)	1.42661	1.46449	1.43906	1.45726	1.43215	1.53462	1.45286
(9, 9)	1.42619	1.46396	1.44096	1.46161	1.43112	1.53216	1.45533
(10, 10)	1.42896	1.46267	1.44216	1.45984	1.43322	1.53891	1.45568
(4, 0)	1.45686	1.45818	1.46311	1.46401	1.48017	1.51946	1.47081
(5, 0)	1.44741	1.48312	1.46043	1.46102	1.48332	1.51785	1.46870
(6, 0)	1.42740	1.46661	1.46028	1.46053	1.48257	1.51965	1.46855
(7, 0)	1.42826	1.46642	1.45967	1.46400	1.48131	1.52311	1.46991
(8, 0)	1.43276	1.47047	1.46025	1.46035	1.48113	1.51917	1.46827
(9, 0)	1.43149	1.46887	1.46085	1.46643	1.48171	1.51322	1.47041
(10, 0)	1.42905	1.46762	1.45951	1.46731	1.48723	1.52113	1.47156
(11, 0)	1.43454	1.46645	1.46055	1.46733	1.48212	1.51753	1.47112
(4, 2)	1.43516	1.47324	1.44182	1.45696	1.44643	1.52073	1.45831
(6, 3)	1.42909	1.46595	1.44544	1.46231	1.44787	1.51176	1.46040
(8 4)	1.42600	1.46394	1.44131	1.45881	1.44787	1.51176	1.45785
(10, 5)	1.42567	1.46292	1.44165	1.46046	1.44906	1.52371	1.46034
(12, 6)	1.42550	1.46598	1.46323	1.46423	1.44917	1.51218	1.46728
(14, 7)	1.43516	1.47324	1.46112	1.46457	1.45038	1.52134	1.46815
(16, 8)	1.42741	1.46295	1.44676	1.46105	1.45091	1.52243	1.46244
(18, 9)	1.42646	1.46393	1.44001	1.45796	1.46333	1.52322	1.46175
(20, 10)	1.42774	1.46297	1.44156	1.45973	1.45357	1.51823	1.46024
			Total average bond length^╂^				1.46168

╂: Total average bond length and average bond length refers to the average bond length of C_0·5_/(BN)_0.5_ heterojunction SWNTs.

**Table 3a:** Average bond angle of SWCNTs and SWBNNTs.

(*n*, *m*)			
(3, 3)	118.2205	118.5492	115.8057
(4, 4)	118.3102	119.1339	117.7787
(5, 5)	118.8572	119.4343	118.2623
(6, 6)	119.2494	119.5727	118.2982
(7, 7)	119.2641	119.5963	118.3257
(8, 8)	119.3918	119.6168	118.3361
(9, 9)	119.6619	119.6307	118.3471
(10, 10)	119.7261	119.6414	118.359
(4, 0)	115.3056	114.3025	113.8568
(5, 0)	117.0753	118.0972	115.2148
(6, 0)	117.8522	118.5932	116.4933
(7, 0)	118.3942	119.4713	117.3756
(8, 0)	118.7532	119.5173	117.9869
(9, 0)	119.0152	119.2967	118.3995
(10, 0)	119.1799	119.4291	118.6964
(11, 0)	119.3403	119.6138	118.9236
(4, 2)	117.1694	118.5705	115.4062
(6, 3)	118.7186	119.5737	117.9478
(8 4)	118.7692	119.6289	118.0035
(10, 5)	119.5315	119.7149	118.2268
(12, 6)	119.6766	119.7767	118.2959
(14, 7)	119.7051	119.7957	118.3872
(16, 8)	119.7349	119.8242	118.5275
(18, 9)	119.7628	119.8604	118.6016
(20, 10)	119.7755	119.8915	118.7653

**Table 3b:** Average bond angle of C_0·5_/(BN)_0.5_ heterojunction SWNTs.

(*n*, *m*)							
(3, 3)	116.3238	118.3375	115.7660	118.2699	115.3953	114.2995	117.5865
(4, 4)	117.8886	118.6302	117.3106	118.8607	117.2820	117.1733	118.5124
(5, 5)	118.6153	118.7552	118.2022	118.8686	118.1503	118.3685	119.0347
(6, 6)	118.9321	118.8988	118.7199	119.3392	118.9461	118.9435	119.3383
(7, 7)	119.1545	119.0291	119.0878	119.7679	119.2283	119.2626	119.5267
(8, 8)	119.2469	119.1344	119.2233	119.8252	119.3405	119.4503	119.6459
(9, 9)	119.3648	119.1968	119.3132	119.8642	119.5321	119.5766	119.7959
(10, 10)	119.5496	119.2221	119.3577	119.8912	119.6225	119.6601	119.7840
(4, 0)	116.0336	117.7962	111.7375	116.7214	114.5686	112.7916	116.1411
(5, 0)	117.3126	117.9723	115.5728	117.1592	115.9979	114.4601	117.0516
(6, 0)	117.3668	118.9147	115.6789	117.8732	117.1187	115.4197	117.8768
(7, 0)	117.4382	118.9958	116.0949	118.6214	117.3023	116.5637	118.0613
(8, 0)	118.0961	119.4986	119.0108	119.2316	117.7936	118.0927	118.6790
(9, 0)	118.7390	119.5733	119.1856	119.3838	117.9713	118.4811	118.9055
(10, 0)	119.5296	119.6034	118.9050	119.5002	118.5296	118.7041	119.1377
(11, 0)	119.6476	119.6903	119.2358	119.7779	118.8169	119.0352	119.2410
(4, 2)	116.0913	119.0133	115.7945	118.3768	116.4757	114.3940	117.5299
(6, 3)	118.3372	119.1686	118.0248	119.5000	117.7738	116.7417	118.6863
(8 4)	119.3007	119.2817	118.7664	119.7213	118.6958	117.8811	119.2617
(10, 5)	119.3707	119.4006	119.0737	119.8092	119.1274	119.5354	119.5127
(12, 6)	119.4773	119.4754	119.1809	119.9187	119.2457	119.5988	119.6532
(14, 7)	119.5821	119.5683	119.2616	119.9258	119.3291	119.7198	119.6984
(16, 8)	119.6420	119.7292	119.3991	119.9323	119.4816	119.7997	119.8943
(18, 9)	119.6840	119.7765	119.4725	119.9704	119.6698	119.8596	119.9296
(20, 10)	119.7484	119.8502	119.6866	119.9801	119.7013	119.9502	119.9344

#### 3.1.2 Structural stability

Although the stability of the structure of $C_{0.5}/{\left(\text{BN}\right)}_{0.5}$ heterojunction SWNTs has been investigated in the literature [39] using molecular dynamics methods, it was obtained that the structure was not only stable, but its stability was comparable to that of SWCNTs. However, $C_{0.5}/{\left(\text{BN}\right)}_{0.5}$ heterojunction SWNTs have not been prepared so far. Therefore, it is necessary to further discuss the stability of its structure.

The most direct and effective way to determine the structural stability of a new material, apart from its molecular dynamical fractionation, is to calculate its phonon spectrum. However, as with molecular dynamics methods, the calculation of phonon spectra requires huge amounts of time. Although the magnitude of the binding energy cannot directly determine whether the structure is stable or not, it is still very effective in determining the relative stability of the structure. For example, for two homogeneous heterogeneous materials, the higher the binding energy is, the better the stability is. Therefore, this paper uses a comparative binding energy approach to partition the structural stability of $C_{0.5}/{\left(\text{BN}\right)}_{0.5}$ heterojunction SWNTs. The binding energy is defined as:

(2) $$E_{b}=\frac{pE\left(\text{atom}\right)-E\left(S\text{WNTs}\right)}{p}$$

In equation (2), the $E_{b}$ is the average binding energy of SWNTs, p is the total number of atoms in the smallest repeating unit of SWNTs, E(atom) is the energy of an individual atom and E(SWNTs) is the energy of the smallest repeating unit of SWNTs.

**Fig. 2:**
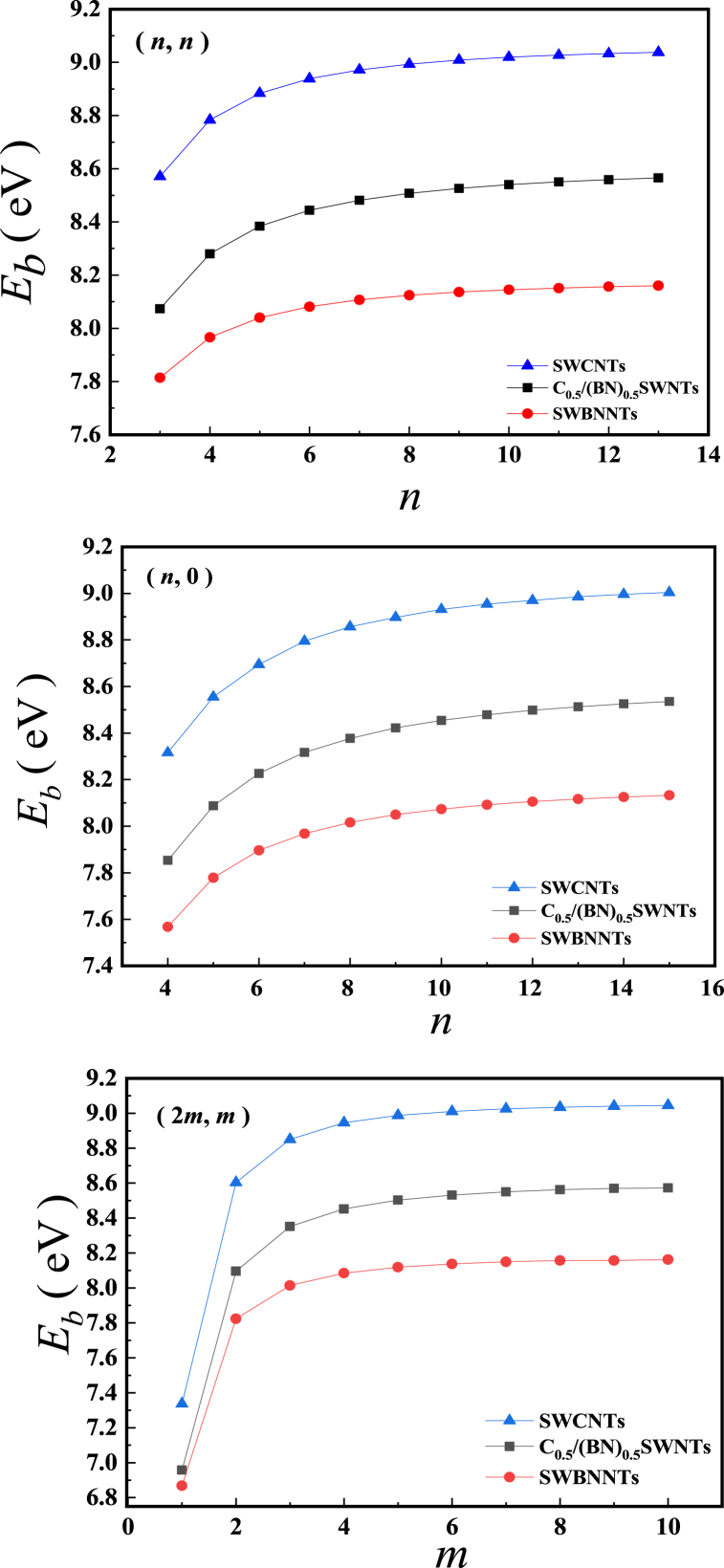
Average binding energies of (*n*, *n*), (*n*, 0) and (2 *m*, *m*).

Fig. 2 shows Average binding energies of (*n*, *n*), (*n*, 0) and (2 *m*, *m*). The binding energy calculations show that, in agreement with the conclusions of the literature [39], for the same *n*, *m*, the binding energy is greatest for SWCNTs, followed by $C_{0.5}/{\left(\text{BN}\right)}_{0.5}$ heterojunction SWNTs, and least for SWBNNTs, indicating that the structure of $C_{0.5}/{\left(\text{BN}\right)}_{0.5}$ heterojunction SWNTs is less stable than SWCNTs but better than SWBNNTs. In particular, the SWBNNTs have been successfully prepared. Therefore, the structural stability of the $C_{0.5}/{\left(\text{BN}\right)}_{0.5}$ heterojunction SWNTs is not in question. It can be said with certainty that with the right approach it is highly likely that $C_{0.5}/{\left(\text{BN}\right)}_{0.5}$ heterojunction SWNTs of all types and tube diameters can be prepared.

### 3.2 Electronic properties and their regulation

#### 3.2.1 Energy band structure and electronic density of states

**Fig. 3:**
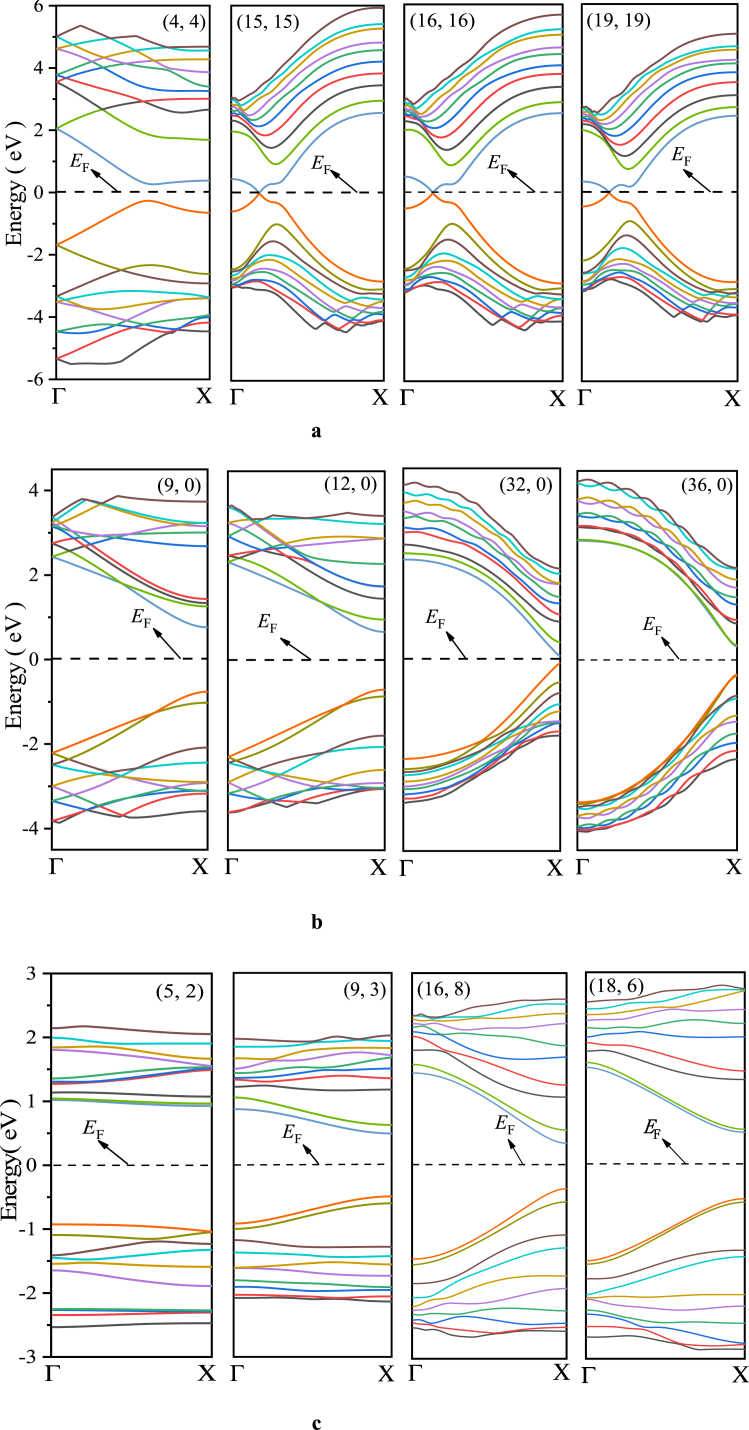
(a) Energy band structure of (4), (4) (b) Energy band structure of (9, 0), (12, 0), (32, 0) and (36, 0) (c) Energy band structure of ([2,5]), ([3,9]), ([8,16]) and ([6,18]).

**Fig. 4:**
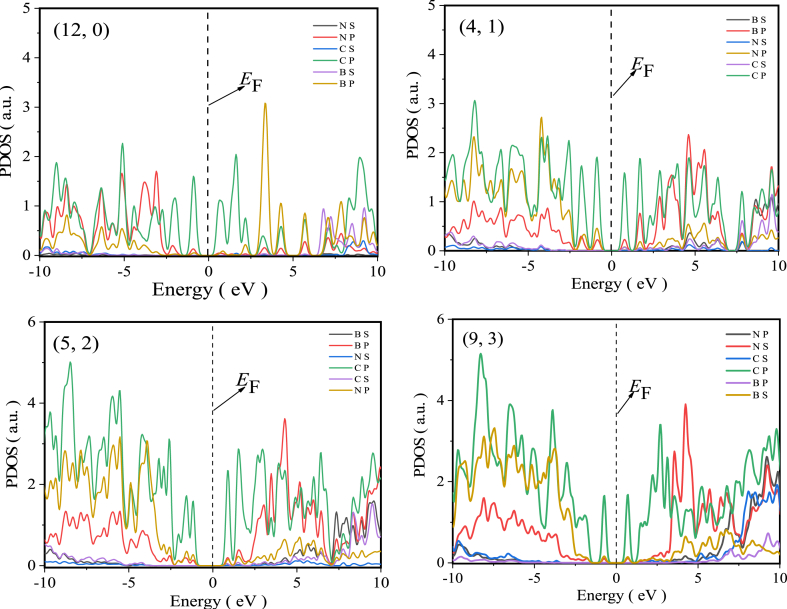
Electronic density of states of ([12], 0), ([1,4]), ([2,5]) and ([3,9]).

In this paper, the band structure of three types of $C_{0.5}/{\left(\text{BN}\right)}_{0.5}$ heterojunction SWNTs (hereinafter denoted as (n,m)) is simulated using 240 K-points and ten energy levels above and below the Fermi level. At the same time, the electronic density of states for the energy band structure is calculated. The results of the energy band structure calculations show (see Fig. 3a–c) that the armchair type (n,n) exhibits the zero band gap structure of metallic conductors and clearly retains the Dirac cone feature when the tube diameter is large (see Fig. 3a); the chiral type (n,m) exhibits the forbidden band structure feature (see Fig. 3c); while the zigzag type (n,0) also exhibits the forbidden band structure feature with the exception of a few (see Fig. 3b). The electronic density of states calculations further shows (see Fig. 4, where the half-height width is 2.5) that the conduction band bottoms and valence band tops of each semiconducting (n,m) are occupied by p-orbital electrons of C atoms, indicating that the heterojunction does open the band gap of non-armchair metallic SWCNTs.

#### 3.2.2 Regulation relation between band gap and indirect-direct properties and diameter and type

In this paper, the regulation of the (n,m) band gap and indirect-direct properties in relation to the tube diameter and type is studied more systematically. The tube diameter *d* is related to *n* and *m* as:

(3) $$d=\frac{\left|{\vec{C}}_{nm}\right|}{\pi }=\frac{\left|{\vec{a}}_{1}\right|}{\pi }{\left(n^{2}+nm+m^{2}\right)}^{\frac{1}{2}}$$

**Fig. 5:**
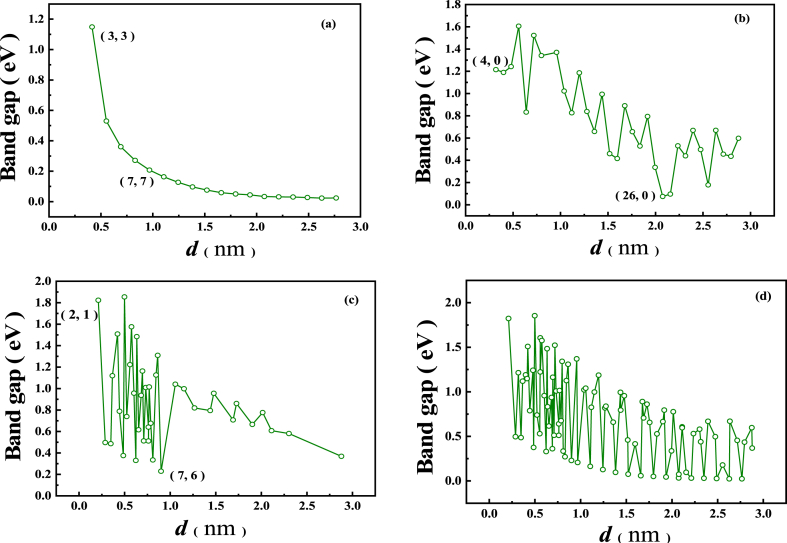
Band gap change curve with tube diameter of the three types and indistinguish C_0·5_/(BN)_0.5_ heterojunction SWNTs (a) (*n*, *n*); (b) (*n*, *0*); (c) (*n*, *m*) *n*≠0, *m*≠0 and *n*≠*m*; (d) indistinguish type (*n*, *m*).

In equation (3), the $\left|{\vec{a}}_{1}\right|=\left|{\vec{a}}_{2}\right|=\sqrt{3}a=2.53$ Å, a(1.46 Å) is the total average bond length of C_0·5_/(BN)_0.5_ heterojunction SWNTs (see Table 2). As shown in Fig. 5, over the range of tube diameters covered in this paper (0.2112–2.8784 nm), the overall band gap oscillates down with increasing tube diameter (see Fig. 5d), with a maximum peak of 1.854eV. Unlike the SWCNTs, all (n,0) are non-metallic semiconductors except for *n* = 26, 27, 32 (band gap <0.200eV) (see Fig. 5b). In agreement with the conclusions of the literature [20], the semiconducting (n,0) have direct band gaps (see Table 4). The chiral type (n,m), on the other hand, all are semiconductor (see Fig. 5c and Table 4) and have both indirect and direct band gaps, with the direct band gaps being the most common (see Table 4). Particularly, it should be noted that the (n,m) band gap maximum is 1.854eV. This is more than 50% than the SWCNTs band gap maximum of 1.2eV, and the non-maximum band gap increases significantly. Of these, (n,n) also has five of the smaller tube diameters which turn out to be non-metallic semiconductors (Qualitatively consistent with literature [38] findings.), with (3), (3) -(6, 6) having an indirect band gap and (7, 7) a direct band gap (See Fig. 5a and Table 4). The aim of opening the band gap of metallic SWCNTs and increasing the band gap of semiconducting SWCNTs while reducing the band gap of SWBNNTs has thus been largely achieved. The results of the above studies show that (n,m) is both the same and different from SWCNTs in terms of electronic properties. Like SWCNTs, (n,m) can be either a metallic conductor or a non-metallic semiconductor. The difference is reflected in the fact that all SWCNTs are metallic conductors when |n−m|=3q(q=1,2,3...). In contrast, (n,m) remains a metallic conductor only when q=0 (except for n≤7); when q≠0, both chiral (n,m) and (n,0) are non-metallic semiconductors. It can be seen, compared with SWCNTs, that the band gap of metallic tubes is almost completely opened, except for (n,n), and the band gap of most of the semiconducting tubes is significantly increased. At the same time, the band gap of the semiconducting (n,m) is generally significantly reduced compared with SWBNNTs, and there is both an indirect band gap and a direct band gap. Particularly, as with SWCNTs, the electronic properties of (n,m) are dual in nature and can be effectively modulated by type and tube diameter control alone. It can be said that (n,m) is a nanomaterial with superior electronic properties compared with SWCNTs and SWBNNTs.

**Table 4:** Band gap and inter direct properties of (*n*, *m*) for semiconductor properties.

(*n*, *m*)	Band gap (eV)	Direct or indirect	(*n*, *m*)	Band gap (eV)	Direct or indirect
(4, 0)	1.214506	Direct	(3, 2)	0.485845	Direct
(5, 0)	1.189832	Direct	(4, 1)	1.119083	Indirect
(6, 0)	1.241803	Direct	(4, 2)	1.508684	Indirect
(7, 0)	1.605669	Direct	(5, 1)	0.788017	Indirect
(8, 0)	0.832411	Direct	(4, 3)	0.375414	Indirect
(9, 0)	1.522446	Direct	(5, 2)	1.853994	Indirect
(10, 0)	1.341387	Direct	(6, 1)	0.739225	Direct
(11, 0)	0.936142	Direct	(5, 3)	1.2228218	Direct
(12, 0)	1.369771	Direct	(6, 2)	1.575613	direct
(13, 0)	1.022237	Direct	(7, 1)	0.956499	Direct
(14, 0)	0.826319	Direct	(5, 4)	0.329952	Direct
(15, 0)	1.186083	Direct	(6, 3)	1.483807	Direct
(16, 0)	0.838399	Direct	(7, 2)	0.615411	Direct
(17, 0)	0.658809	Direct	(8, 1)	0.936324	Direct
(18, 0)	0.993939	Direct	(6, 4)	1.163246	Direct
(19, 0)	0.458968	Direct	(7, 3)	0.511455	Direct
(20, 0)	0.415382	Direct	(8, 2)	1.009101	Indirect
(21, 0)	0.890091	Direct	(9, 1)	0.640127	Indirect
(22, 0)	0.656443	Direct	(6, 5)	0.511139	Indirect
(23, 0)	0.527578	Direct	(7, 4)	1.014674	Direct
(24, 0)	0.794342	Direct	(8, 3)	0.676321	Direct
(25, 0)	0.336603	Direct	(9, 2)	0.334204	Direct
(28, 0)	0.530337	Direct	(8, 4)	1.126334	Direct
(29, 0)	0.439164	Direct	(9, 3)	1.309194	Direct
(30, 0)	0.668919	Direct	(7, 6)	0.229177	Indirect
(31, 0)	0.496244	Direct	(10, 5)	1.040244	Direct
(33, 0)	0.669431	Direct	(12, 4)	0.997788	Direct
(34, 0)	0.455147	Direct	(12, 6)	0.819428	Direct
(35, 0)	0.435401	Direct	(15, 5)	0.79448	Direct
(36, 0)	0.597131	Direct	(14, 7)	0.955343	Direct
(3, 3)	1.148769	Indirect	(16, 8)	0.707125	Direct
(4, 4)	0.529889	Indirect	(18, 6)	0.86069	Direct
(5, 5)	0.360711	Indirect	(18, 9)	0.665714	Direct
(6, 6)	0.271251	Indirect	(21, 7)	0.776442	Direct
(7, 7)	0.207285	Direct	(20, 10)	0.607061	Direct
(2, 1)	1.823184	Indirect	(24, 8)	0.580709	Direct
(3, 1)	0.496244	Direct	(30, 10)	0.368317	Direct

### 3.3 Controllable preparation

The literature [39] and the comparative studies of binding energy in this paper show that the structure of (n,m) is stable and must be able to be prepared. To date, however, there have been no reports of its successful preparation and the point at issue is the preparation method. Due to the specificity of the structure, it is clearly undesirable to adopt the pore popular such as bottom-up, zero-dimensional to one-dimensional, i.e., nanotube preparation from quantum dots by layer-by-layer growth methods.

**Fig. 6:**
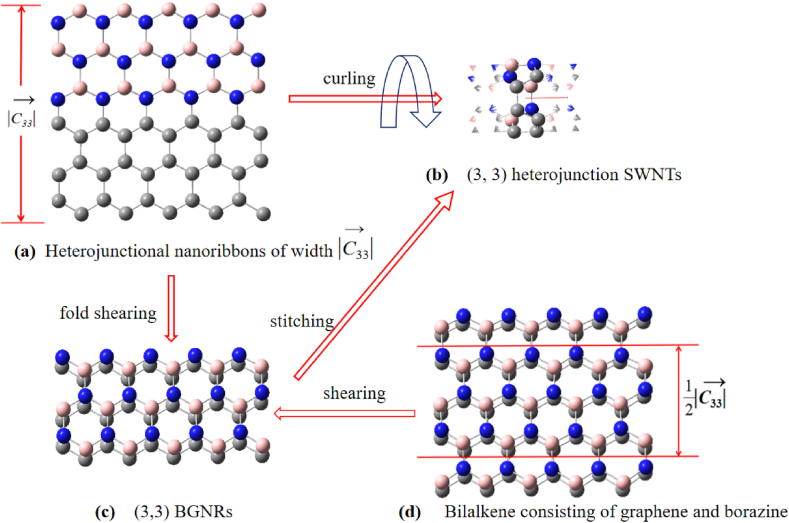
Illustration of the interrelationship between the wide $\left|{\vec{C}}_{33}\right|$**heterojunction nano bands (a), (3, 3) heterojunction SWNTs (b), (3, 3) BGNRs (c) and bilalkene consisting of graphene and borazine (d)**.

**Fig. 7:**
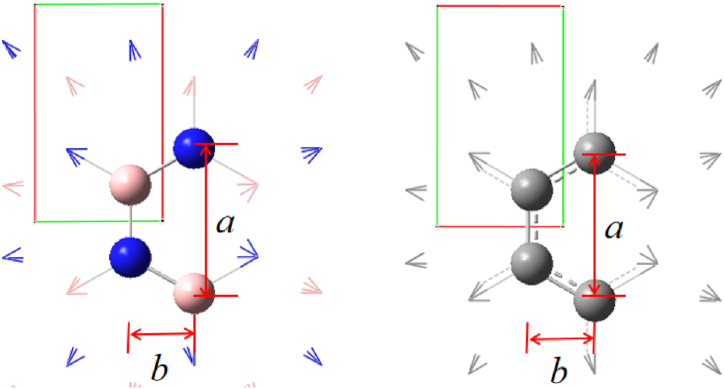
Borazene and graphene crystals and lattice constants.

The reason for this is that the layer-by-layer growth process, with C and BN dividing the tube wall equally along the mother line, is so difficult to manipulate that it is almost impossible to achieve half of the tube wall for each C and BN. Considering that, like non-heterojunction SWCNTs or SWBNNTs, (n,m) can also be considered to be formed by curling nanoribbons of $\left|{\vec{C}}_{nm}\right|$ width with the modal length of their chiral vector ${\vec{C}}_{nm}$ as the perimeter (see Fig. 6a and b) [39]. The difference is that in this case the nanoribbons are a combination of carbon nanoribbons and boron nitride nanoribbons of 1/2 $\left|{\vec{C}}_{nm}\right|$ the width in the form of a heterojunction (see Fig. 6a). In the literature [46,47], SWCNTs and SWBNNTs were successfully prepared by shearing two layers of graphite and double layer borazene (also known as Bilayer h-BN), respectively, using the “electron beam cutting” method. The biggest advantage of the “electron beam cutting” method is that it is easier to prepare small diameter SWNTs, and this approach is also the basis of the theory study of controlled preparation in this paper. Based on the literature [46,47], our research group has designed a method for the controllable preparation of single-walled silicon carbide nanotubes, the “selective shear slit method” [48], which is innovative in that by selectively shearing the bilalkene, a bilayer nanoribbon satisfying the folded relationship (see Fig. 6 a and c) is obtained, enabling the controlled preparation of various classes of non-heterojunction SWNTs by the “electron beam cutting method”, i.e., the controllable preparation of various classes of non-heterojunction SWNTs according to the size of the band gap and the indirect-direct properties. Since there is only a small lattice mismatch between Graphene (a layer of graphite) and borazene (a layer of h-BN) (see Table 5 and Fig. 7), it is possible to use the “selective shear stitching method” to shear bilayers of Graphene and boron nitride to obtain bilayers of carbon and boron nitride nanoribbons that satisfy the fold relationship (see literature [48] for how to ensure that the fold relationship is satisfied), referred to as bilayers of (n,m) BGNRs (see Fig. 6a, c and d). The question now is whether it is possible to stitch the (n,m) BGNRs along the shear edges to finally obtain (n,m) (see Fig. 6 c and b). To this end, computer simulations of the stitching of (n,m) BGNRs into tubes along shear edges are carried out and their corresponding formation and activation energies are calculated. The formation energy is defined as:

(4) $$E_{f}=\frac{E_{T}\left(\text{SWNTs}\right)-E_{T}\left(\text{BGNRs}\right)}{q}$$

(5) $$E_{T}\left(\text{BGNRs}\right)=E_{T}\left(\text{up}\right)+E_{T}\left(\text{down}\right)$$

In equations (4), (5), the $E_{T}\left(\text{SWNTs}\right)$ is the energy of the (n,m) smallest repeating unit, $E_{T}\left(\text{BGNRs}\right)$ is the energy of the smallest repeating unit of BGNRs, and q is the number of atoms in the BGNRs or (n,m) smallest repeating unit. $E_{T}\left(\text{up}\right)$ and $E_{T}\left(\text{down}\right)$ denote the energy of the smallest repeating unit of borazene nanoribbons and graphene nanoribbons, respectively.

**Table 5:** Borazene and graphene lattice constants and relative differences.

Lattice constants	Borazene	Graphene	Borazene relative differences（%）	Graphene relative differences（%）
a	2.92334	2.84904	2.541	2.608
b	1.26449	1.23359	2.444	2.505

Activation energy is defined as:

(6) $$E_{a}=\frac{E_{b}-E_{T}\left(\text{BGNRs}\right)}{q}$$

(7) $${E_{a}}^{\prime }=\frac{E_{b}-E_{T}\left(\text{SWNTs}\right)}{q}$$

In equations (6), (7), the $E_{a}$ denotes the activation energy of the positive reaction process, i.e., the average heat of reaction from the BGNRs to the transition state; ${E_{a}}^{\prime }$ denotes the activation energy of the negative reaction process, i.e., the average heat of reaction from the transition state to the SWNTs; and $E_{b}$ denotes the energy of the smallest repeating unit of the transition state.

**Fig. 8:**
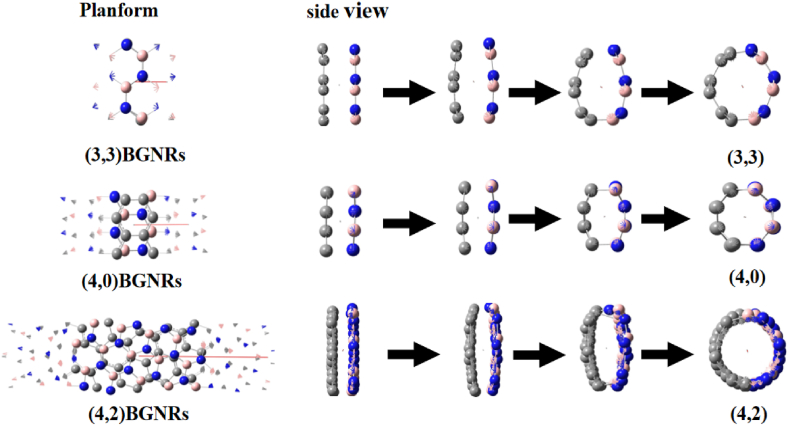
Stitching from (3), (3) BGNRs, (4, 0) BGNRs and (4, 2) BGNRs to (3, 3), (4, 0) and (4, 2) into tubes.

**Fig. 9:**
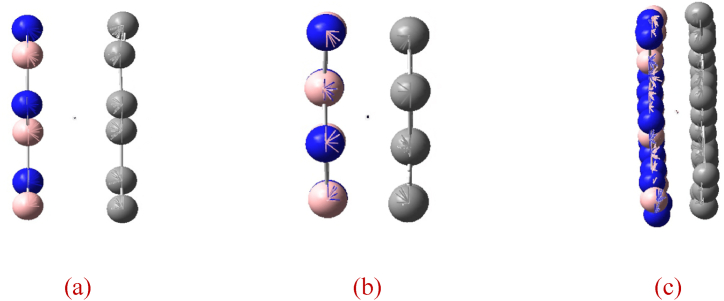
The dynamic computer simulation (a), (b), (c) from (3), (3) BGNRs, (4, 0) BGNRs and (4, 2) BGNRs to (3, 3), (4, 0) and (4, 2) in stitching Comment: If these motion picture cannot be viewed, please download it in the supplementary material.

**Fig. 10:**
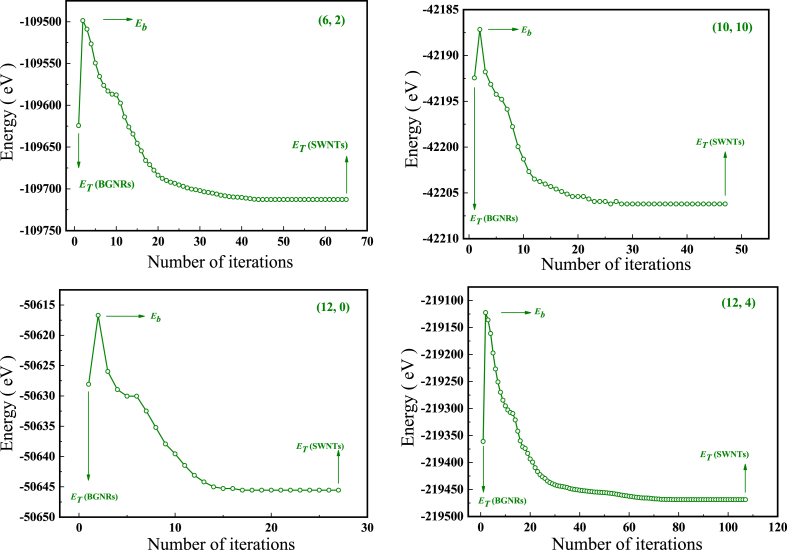
Energy change from (6, 2) BGNRs, (10, 10) BGNRs, (12, 0) BGNRs and (12, 4) BGNRs to (6, 2), (10, 10), (12, 0) and (12, 4) in stitching process.

The computer simulation results of the tube-forming process showed that (n,m) was sutured from (n,m) BGNRs, which was a self-organizing process without human intervention (see Fig. 8, Fig. 9). The formation energy calculations show that the transition from (n,m) BGNRs to (n,m) is a possible exothermic process from a high energy state to a low energy state (see Fig. 10 and Table 6). Whereas the calculated activation energies are either negative or, to a lesser extent positive value (see Fig. 10 and Table 7), it is further shown that it is not only possible but feasible to obtain (n,m) by stitching the (n,m) BGNRs.

**Table 6:** Different species (*n*, *m*) form the energy.

(*n*, *m*)	*E*_*T*_ (up) (eV)	*E*_*T*_ (down) (eV)	*E*_*T*_ (SWNTs)	*q*	*E*_*T*_ (formation) (eV/atom)
(3, 3)	−6465.425	−6180.681	−12656.309	12	−0.850242
(4, 4)	−8624.332	−8243.566	−16878.376	16	−0.654855
(5, 5)	−10782.243	−10306.589	−21100.039	20	−0.562391
(6, 6)	−12940.126	−12369.066	−25321.484	24	−0.512108
(7, 7)	−14428.332	−15095.778	−29538.181	28	−0.502593
(8, 8)	−17255.227	−16494.717	−33764.027	32	−0.440159
(10, 10)	−21571.376	−20621.071	−42206.326	40	−0.346964
(4, 0)	−8621.704	−8240.031	−16871.542	18	−0.544895
(5, 0)	−10779.813	−10303.044	−21094.115	20	−0.562932
(6, 0)	−12935.272	−12365.830	−25316.265	24	−0.631828
(7, 0)	−15095.135	−14428.493	−29538.181	28	−0.519725
(8, 0)	−17253.774	−16491.535	−33759.856	32	−0.454630
(9, 0)	−19411.926	−18554.575	−37981.464	36	−0.415501
(10, 0)	−21569.849	−20617.484	−42202.931	40	−0.389166
(11, 0)	−23727.722	−22680.470	−46424.263	44	−0.365226
(12, 0)	−25885.422	−24742.657	−50645.586	48	−0.364741
(4, 1)	−45258.976	−43262.185	−88586.852	84	−0.782075
(4, 2)	−30164.237	−28836.352	−59063.999	56	−1.132466
(5, 1)	−68975.958	−61807.993	−130887.141	124	−0.832208
(5, 2)	−84078.136	−80358.871	−164557.143	156	−0.770102
(6, 2)	−56055.341	−53568.958	−109712.861	104	−0.851635
(6, 3)	−45268.066	−43264.759	−88617.509	84	−1.008151
(8, 4)	−60362.311	−57674.815	−118167.913	112	−1.167731
(9, 3)	−81941.935	−82429.599	−164497.481	156	−0.807391
(10, 5)	−73296.071	−74217.341	−147621.824	140	−0.774373
(12, 4)	−112156.331	−107204.644	−219468.873	208	−0.518703

**Table 7:** Activation energy for different species (*n*, *m*).

(*n*, *m*)	*E*_*T*_ (BGNRs) (eV)	*E*_*b*_ (eV)	*E*_*T*_ (SWNTs) (eV)	*q* (atom)	*E*_*a*_ (eV/atom)	*E*_*a*_′ (eV/atom)
(3, 3)	−12646.107	−12647.033	−12656.309	12	−0.077171	0.773071
(4, 4)	−16867.898	−16867.409	−16878.376	16	0.030622	0.685456
(5, 5)	−21088.792	−21087.703	−21100.039	20	0.054386	0.616779
(6, 6)	−25309.194	−25309.059	−25321.484	24	0.005592	0.517700
(7, 7)	−29524.109	−29525.163	−29538.181	28	−0.037682	0.464912
(8, 8)	−33749.942	−33750.274	−33764.027	32	−0.010395	0.429763
(10, 10)	−42192.448	−42187.162	−42206.326	40	0.132121	0.479085
(4, 0)	−16861.735	−16856.089	−16871.5426	18	0.313624	0.858519
(5, 0)	−21082.857	−21078.343	−21094.1155	20	0.225681	0.788619
(6, 0)	−25301.102	−25292.76	−25316.2657	24	0.347577	0.979405
(7, 0)	−29523.629	−29516.183	−29538.181	28	0.265886	0.785611
(8, 0)	−33745.309	−33739.934	−33759.8568	32	0.167948	0.622579
(9, 0)	−37966.502	−37954.704	−37981.4602	36	0.327702	0.743203
(10, 0)	−42187.334	−42179.271	−42202.9006	40	0.201558	0.590725
(11, 0)	−46408.191	−46401.389	−46424.2607	44	0.154576	0.519802
(12, 0)	−50628.079	−50616.704	−50645.5866	48	0.236975	0.601715
(4, 1)	−88521.156	−88439.162	−88586.8506	84	0.976123	1.758198
(4, 2)	−59000.582	−58971.959	−59063.9998	56	0.511129	1.643595
(5, 1)	−130783.951	−130642.652	−130887.144	124	1.139508	1.971716
(5, 2)	−164437.007	−164390.477	−164557.142	156	0.298266	1.068368
(6, 2)	−109624.299	−109498.595	−109712.869	104	1.208712	2.060348
(6, 3)	−88532.825	−88435.08	−88617.5098	84	1.163630	2.171781
(8, 4)	−118037.127	−117918.882	−118167.912	112	1.055751	2.223483
(9, 3)	−164371.535	−164203.535	−164497.488	156	1.076921	1.884313
(10, 5)	−147513.412	−147362.684	−147621.824	140	1.076631	1.851005
(12, 4)	−219360.983	−219122.398	−219468.873	208	1.147232	1.665935

## 4 Conclusion

In summary, the structure of $C_{0.5}/{\left(\text{BN}\right)}_{0.5}$ heterojunction SWNTs is stable. Their electronic properties can be either metallic conductors or direct or indirect second-generation semiconductors with very good performance, and the direct or indirect nature and size (0–1.854eV) can be tuned in the range of 0.4–2.2 nm tube diameters. In particular, the simulation of the dynamic processes and formation and activation energies of $C_{0.5}/{\left(\text{BN}\right)}_{0.5}$ heterojunction SWNTs obtained from BGNRs via stitching show that the tunable preparation of $C_{0.5}/{\left(\text{BN}\right)}_{0.5}$ heterojunction SWNTs can be achieved using electron-beam dicing, i.e., using a top-down, two-dimensional to (quasi-)one-dimensional approach, with type and tube diameter controlling.

## Declarations

### Author contribution statement

Feiyu Zhu: Performed the experiments; Analyzed and interpreted the data; Wrote the paper.

Yanbo Zou: Conceived and designed the experiments; Contributed reagents, materials, analysis tools or data.

Junzhe Lu: Conceived and designed the experiments.

Jie Wei: Analyzed and interpreted the data.

Hengjiang Zhu: Conceived and designed the experiments; Analyzed and interpreted the data.

### Data availability statement

All data to support the conclusions have been either provided or are otherwise publicly available.

## Declaration of competing interest

The authors declare that they have no known competing financial interests or personal relationships that could have appeared to influence the work reported in this paper.
